# 

**Published:** 1881-11-20

**Authors:** 


### TABLE OF CONTENTS.

ORIGINAL AND SELECTED ARTICLES.

The Nature, Pathology and Treatment of Dipsomania, by Edward C. Mann, M. D., of New York, 401. Some of the uses of Nitro-Glycerine, by William A. Hammond, M. D., of New York City, 407. Abdominal Surgery and Listerism, 409. Black Haw, by Robert Boal, M.D., of Peoria, Ill., 412. Incised wound of Intestine—Recovery, 413. Recurrent or Obstinate Malarial Attacks, by John H. Pool, M.D., South Mills, N. C.; Camphor and Hydrate Chloral, 415. Obstetric Aphorisms, by H. Webster Jones, M. D., Chicago, 416.

ABSTRACTS AND GLEANINGS.

Is Guiteau Insane, 417. Treatment of Intermittent and Remittent Fever, 418. Treatment of Nasal Catarrh, 420. Acute Rheumatism, 421. Syphilitic Alopecia, 422. Treatment of Typhoid Fever by Salicylate of Soda; Acetic Acid in Small-Pox, 423. Treatment of Epilepsy 424. For Nervous Diarrhœa; Eucalyptus Globulus, 425. New Method of Producing Anæsthesia in the Larynx; Human and Animal Variola, 426. Potassium Bromide in Orchitis and Inflamed Breast; Defer's Method of Treatment of Simple Hydrocele; Male Wet-Nurse, 427. Burns; Protective Syphilization; The Camphor Tree; Eucalyptol in Albuminuria, 428. Bicarbonate of Soda in Tonsilitis; Œsophagoscopes; Chorea of Childhood, 429. Quinine Elimination and Absorption; Tartrate of Quinoline; Effects of Light on the Skin, 430.

SCIENTIFIC ITEMS.

To Hear the Grass Grow; Cotton-Seed Oil; Giants and Dwarfs, 431. Electricity of Rubber; Petroleum on Trees and Bushes; Tincture of Chloride of Iron, 432.

PRACTICAL NOTES AND FORMULÆ.

The Use of Quinine, 433. Antiseptic Inhalation; Hop Bitters; For Dyspepsia, 434. A Combined Stomachic and Laxative; For Gonorrhœa; Purgative Pill, 435.

EDITORIALS AND MISCELLANEOUS.

Read; Dr. Theophilus Parvin; See new advertisement of Kidder & Laird; Walshe's Physicians' Combined Call Book and Tablet; Antiseptic Surgery, 436. The Physician's Hand Book for 1882; The Medical Record Visiting List; Stand from Under; The Doctors of Georgia; Report of Lecture on Ophthalmology and Otology, 437. Book Notices, 438. Special Notices, 440.

